# Minimum Field Strength Simulator for Proton Density Weighted MRI

**DOI:** 10.1371/journal.pone.0154711

**Published:** 2016-05-02

**Authors:** Ziyue Wu, Weiyi Chen, Krishna S. Nayak

**Affiliations:** 1 Ming Hsieh Department of Electrical Engineering, University of Southern California, Los Angeles, California, United States of America; 2 Department of Biomedical Engineering, University of Southern California, Los Angeles, California, United States of America; Brighton and Sussex Medical School, UNITED KINGDOM

## Abstract

**Objective:**

To develop and evaluate a framework for simulating low-field proton-density weighted MRI acquisitions based on high-field acquisitions, which could be used to predict the minimum B_0_ field strength requirements for MRI techniques. This framework would be particularly useful in the evaluation of de-noising and constrained reconstruction techniques.

**Materials and Methods:**

Given MRI raw data, lower field MRI acquisitions can be simulated based on the signal and noise scaling with field strength. Certain assumptions are imposed for the simulation and their validity is discussed. A validation experiment was performed using a standard resolution phantom imaged at 0.35 T, 1.5 T, 3 T, and 7 T. This framework was then applied to two sample proton-density weighted MRI applications that demonstrated estimation of minimum field strength requirements: real-time upper airway imaging and liver proton-density fat fraction measurement.

**Results:**

The phantom experiment showed good agreement between simulated and measured images. The SNR difference between simulated and measured was ≤ 8% for the 1.5T, 3T, and 7T cases which utilized scanners with the same geometry and from the same vendor. The measured SNR at 0.35T was 1.8- to 2.5-fold less than predicted likely due to unaccounted differences in the RF receive chain. The predicted minimum field strength requirements for the two sample applications were 0.2 T and 0.3 T, respectively.

**Conclusions:**

Under certain assumptions, low-field MRI acquisitions can be simulated from high-field MRI data. This enables prediction of the minimum field strength requirements for a broad range of MRI techniques.

## Introduction

Magnetic resonance imaging (MRI) is one of the most powerful imaging modalities, and has had a significant impact on healthcare [1]. MRI is safe, non-invasive, non-ionizing, and is capable of resolving tissues in three dimensions while providing several different types of tissue contrast in a single examination. MRI has two notable limitations, cost and speed. These are highly relevant in an era where rising healthcare costs [2] have placed greater pressure on determining and optimizing the cost-effectiveness of imaging for specific clinical questions. To date, standard clinical MRI (1.5 T/3 T) has proven to be cost-prohibitive for many potential screening and preventative medicine applications. Even for diagnostic applications, achieving better image quality without improving outcomes, at the expense of reducing access due to high cost, can be only counterproductive [3]. On the other hand, low-field MRI (≤ 0.5 T) can be much less expensive while still maintaining equivalent diagnostic values for certain applications, as demonstrated by Rutt et al. [4].

Several technological developments have helped to address the speed and temporal resolution of MRI scanning. Fast gradients and parallel imaging have had a significant impact and are now available on almost all commercial MRI scanners. Constrained reconstruction [5], compressed sensing [6], and MR fingerprinting [7] are emerging techniques that provide the potential added benefit of de-noising. These technological advances are typically developed and tested first on high-field scanners, defined here as ≥1.5 T.

The purpose of this work is to provide a framework for determining the minimum field strength requirements of novel MRI methods. Due to the difficulties in differentiating different species in k-space, the current framework is most appropriate for proton-density weighted (PDw) acquisitions. Using this tool, researchers could determine the relevance and applicability of their techniques at lower field strengths (e.g. 0.1 to 0.5 T) even if they have only had the opportunities to test them at high field strengths (e.g. ≥1.5 T). When applied to de-noising techniques and constrained reconstruction, this could also enable researchers to determine if their techniques could enable a reduction in the cost of MRI, should such instruments be designed for their applications. In this manuscript, we provide phantom validation of this framework, and provide two illustrative examples of how to predict minimum field strength requirements.

The first example application is real-time upper airway imaging, for the assessment of sleep-disordered breathing. The lack of anatomical information is a major limitation for current sleep studies, and dynamic MRI has been shown [8–11] to be an promising method to fulfill this unmet need. The high cost associated with conventional clinical MRI scans is arguably the number one reason that prevents these methods from being applied to routine sleep studies. If the scans can be performed on low-field scanners at much lower cost, the option of including MRI in sleep studies will be much more realistic. Besides lower cost, reduced Lorentz force experienced by the gradient coils and hence lower acoustic noise is another attractive feature of low-field MRI, especially for imaging during sleep.

The second example application is the measurement of liver fat fraction. Conventional high-field MRI has proven to be a powerful tool for body and organ fat distribution assessment [12,13] and for tissue fat fraction quantification [14]. It has the ability to resolve all fat depots and to measure organ fat. As obesity prevalence continues to rise, there is increasing need of accurate and low cost tools for assessing and quantifying body fat distribution including organ fat. If fat-water separated MRI can be performed at a much lower per-scan-cost, it could become the most cost-effective technique to address body composition assessment in preventative medicine.

## Materials and Methods

### Modeling Assumptions

To simulate low-field acquisition from data acquired at high field strength, we make six assumptions, listed in Table 1, and explained below.

**Table 1:** Assumptions for Low Field Acquisition.

• Body noise dominance
• Consistent RF transmit field ($B_{1}^{+}$)
• Consistent RF receive field ($B_{1}^{-}$) and noise covariance (Σ)
• Consistent *B*_0_ homogeneity
• Single species dominance or PDw
• Steady state acquisition

#### (1) Body noise dominance

We assume that body thermal noise is the dominant noise source at all field strengths under investigation (0.1–3.0 T). The validity of this assumption depends on field strength, imaging volume and the receiver coil. It has been shown that body noise dominance can be achieved at frequencies as low as 4 MHz in system sizes compatible with human extremity [15,16], suggesting the feasibility of performing most human scans with body noise dominance at 0.1 T or above.

#### (2) Consistent $B_{1}^{+}$ field

We assume that the uniformity of RF transmission is consistent across field strengths. Since the RF operating frequencies go down at low field, the flip angle variation is expected to be smaller in real low-field imaging compared to our simulation.

#### (3) Consistent $B_{1}^{-}$ field

We assume that the receiver coils have the same geometry and noise covariance at different field strengths. In order to simulate arbitrary ${\text{B}}_{1}^{-}$ field, it would require accurate coil maps and noise covariance at both acquired and simulated fields, which one may not have.

#### (4) Consistent B_0_ homogeneity

We assume the same off-resonance in parts-per-million (ppm) at different field strengths. This results in less off-resonance in Hz at lower field.

#### (5) Single species dominance or PDw

We use a single global relaxation correction function to account for the signal change at different field strengths. Because it is difficult to separate different species from k-space data, this assumption requires similar relaxation patterns at different field strengths for anything that contributes a significant portion to the signal in the region of interest. Although it may be unrealistic for some applications, this restriction can be relaxed in certain cases. For PDw imaging, the simulation is still valid when multiple major species are present (see Appendix for details).

#### (6) Steady state acquisition

If the signals are not acquired at steady state, the magnetization relaxation will be determined not only by the sequence parameters but also by the initial state. As a result, a single global relaxation correction cannot be applied and a more complicate time-depend function would need to be calculated.

### Simulation of Low Field Acquisition

**Fig 1:**
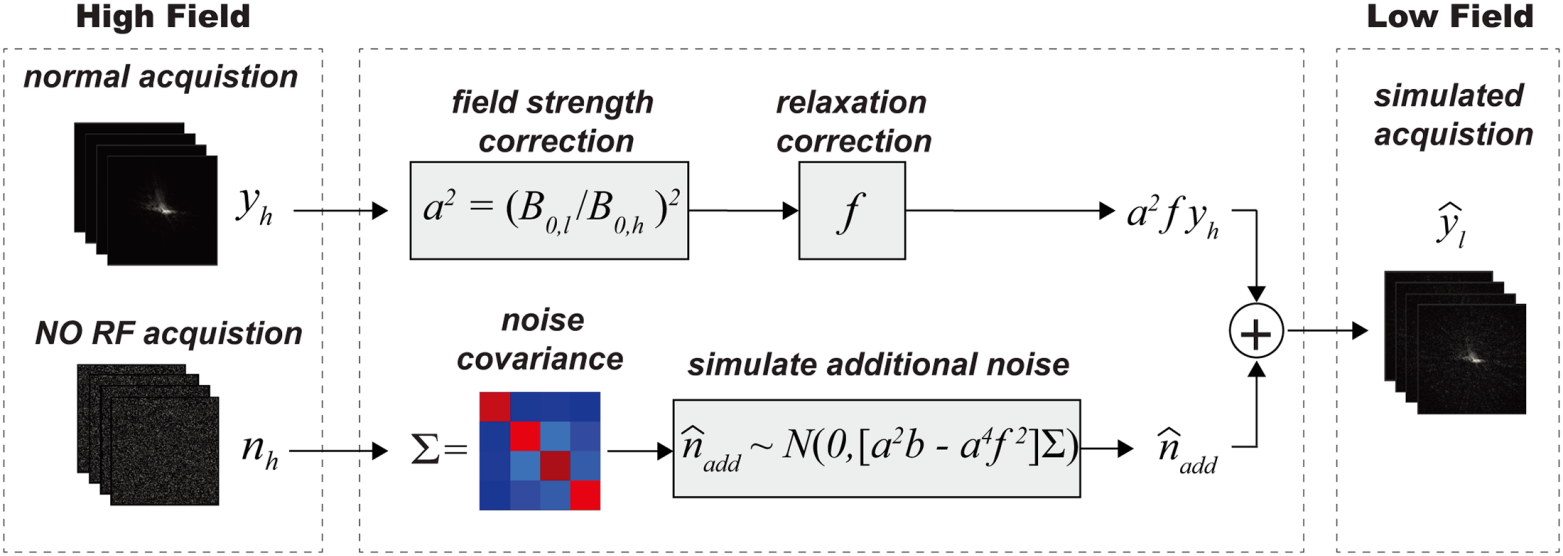
Simulation of Low-field k-Space Data. High-field k-space data *y*_*h*_ and pure noise *n*_*h*_ are first acquired and served as input. *y*_*h*_ is then scaled by *a*^2^ and *f* to account for signal magnitude change and different relaxation behaviors at different field strengths. *f* can be determined based on steady state signal equations for different types of sequences (see Appendix for details). To simulate low-field data ${\hat{y}}_{l},$ additional noise ${\hat{n}}_{add}$, as calculated in the text, is added to compensate for the different noise levels.

The process for simulating low-field data from high-field acquired data is illustrated in Fig 1, and described here. The acquired high-field k-space data can be written as:

$$y_{h}\;=\;s_{h}+n_{h}$$ (1)

Where *s*_*h*_ and *n*_*h*_ are pure signal and noise respectively. Under body noise dominance, both the real and imaginary parts of the k-space noise *n*_*h*_ can be modeled as multivariate normal distributions:

$$Re\left\{n_{h}\right\}\;\sim \;N\left(0,\Sigma \right),\;\;Im\left\{n_{h}\right\}\;\sim \;N\left(0,\Sigma \right)$$ (2)

Where Σ ∈ ℝ^k×k^ is the noise covariance matrix for a k-channel receiver coil and is easily measured by data acquisition with RF turned off. Since the thermal noise variance is proportional to B_0_^2^ and readout bandwidth BW, the simulated noise ${\hat{n}}_{l}$ at low field becomes:

$$Re\left\{{\hat{n}}_{l}\right\}\;\sim \;N\left(0,a^{2}b\Sigma \right),\;\;Im\left\{{\hat{n}}_{l}\right\}\;\sim \;N\left(0,a^{2}b\Sigma \right),\;a=\frac{B_{0,l}}{B_{0,h}},\;b=\frac{BW_{l}}{BW_{h}}$$ (3)

where *l* and *h* stand for low and high field respectively. The pure k-space signal at low field can be modeled as:

$${\hat{s}}_{l}\;=\;a^{2}fs_{h}$$ (4)

Where *f* is a function that represents the signal change due to different relaxation behaviors at different fields. This can be determined with knowledge of the sequence parameters and the dominant species’ relaxation times. The details of calculating *f* for common sequences are provided in the Appendix. Given *f*, the simulated low field k-space data can be written as:

$${\hat{y}}_{l}\;=\;{\hat{s}}_{l}+{\hat{n}}_{l}\;=\;a^{2}fs_{h}+{\hat{n}}_{l}$$ (5)

we can rewrite it as:

$${\hat{y}}_{l}=a^{2}fy_{h}+{\hat{n}}_{add}$$ (6)

Where ${\hat{n}}_{l}={\hat{n}}_{add}+a^{2}fn_{h}$, and from Eqs (2) & (3), we have

$$Re\left\{{\hat{n}}_{add}\right\}\;\sim \;N\left(0,\left(a^{2}b-a^{4}f^{2}\right)\Sigma \right),\;\;Im\left\{{\hat{n}}_{add}\right\}\;\sim \;N\left(0,\left(a^{2}b-a^{4}f^{2}\right)\Sigma \right)$$ (7)

A MATLAB implementation based on the process above as well the examples in this article are available at http://mrel.usc.edu/share.html.

### Phantom Validation

To validate the proposed framework, a standard resolution phantom was scanned using a product sequence on 1.5T, 3T, and 7T whole body scanners, all from the same manufacturer (General Electric, Waukesha, WI). The phantom contains NiCl_2_*H_2_O and H_2_O. T/R birdcage head coils (30cm diameter) were used at all field strengths. The 1.5T and 3T coils were single-channel. The 7T coil has two receive channels with nearly identical sensitivities; data from only one channel was used. We also scanned the same phantom on a 0.35T ViewRay scanner with a 12-channel torso coil. A single image was formed from all channels using sum of square. The scanner has a cylindrical bore similar to the 1.5T/3T/7T scanners, but is manufactured by a different vendor. Due to its primary function as a MRI-guided radiation therapy instrument, the 0.35T scanner has a unique RF coil design to minimally obstruct the radiation source.

Identical acquisition parameters were used on all four scanners: 2D FSPGR with 62.5% partial k-space acquisition; FA 10°; TE/TR 3.1/10 ms; BW 31.25 KHz; FOV 25.6 cm; matrix size 256x160; slice thickness 5 mm. T1 and T2 values were measured using inversion recovery SE and SE sequence respectively. Homodyne reconstruction [17] was performed for all images. SNRs were measured in all cases on the magnitude images. For simulated images, the mean and standard deviation of SNR of twenty different simulations were calculated.

### Real-time Upper Airway Imaging

For sleep apnea patients, airway compliance is measure of muscle collapsibility. This involves ultrafast 2D axial imaging of the airway and simultaneous airway pressure measurement [9]. During the process, negative pressure is generated by briefly blocking inspiration for one to three breaths. Under these circumstances, airway motion is extremely rapid, requiring about 10 frames per second and millimeter resolution. A custom sequence using 2D golden-angle radial FLASH [18] was implemented on the 3T scanner to acquire an oropharyngeal axial slice of one sleep apnea patient with a 6-channel carotid coil. Imaging parameters: 5° flip angle, 6 mm slice thickness, 1 mm^2^ resolution, TE/TR 2.6/4.6 ms, BW 62.5 KHz. A separate scan with RF turned off was performed to calculate the noise covariance. Acquisitions at various low field strengths were simulated using the same imaging parameters.

Twenty-one spokes were used to reconstruct each temporal frame. Conventional gridding [19] was performed on the acquired 3T data and all simulated low-field data. CG-SENSE [20] was also performed with a temporal finite difference sparsity constraint [21]. The NUFFT toolbox [22] was used during algorithm implementation.

### Fat-Water Separation

Fully sampled k-space data were collected using an investigational IDEAL sequence. An 8-channel cardiac receiver coil was used to scan one adult volunteer at 3 T. Slice thickness 5 mm, TE 1.4/2.3/3.2 ms, TR 9 ms, flip angle 3°, BW 62.5KHz. To achieve the same phase shift between fat and water, the product of B_0_ and TE needs to remain the same. Therefore TE’s were set to be (*B*_0,*h*_/*B*_0,*l*_) times longer when simulated at low fields. Bandwidths were also set to (*B*_0,*h*_/*B*_0,*l*_) times shorter, enabled by longer TE’s. Images were reconstructed using the graph cut field-map estimation method [23] from the ISMRM fat-water toolbox [24].

All studies involved were approved by the Institutional Review Board of Children's Hospital at Los Angeles and University of Southern California. Written informed consents were obtained from the participants.

## Results

### Phantom validation

**Fig 2:**
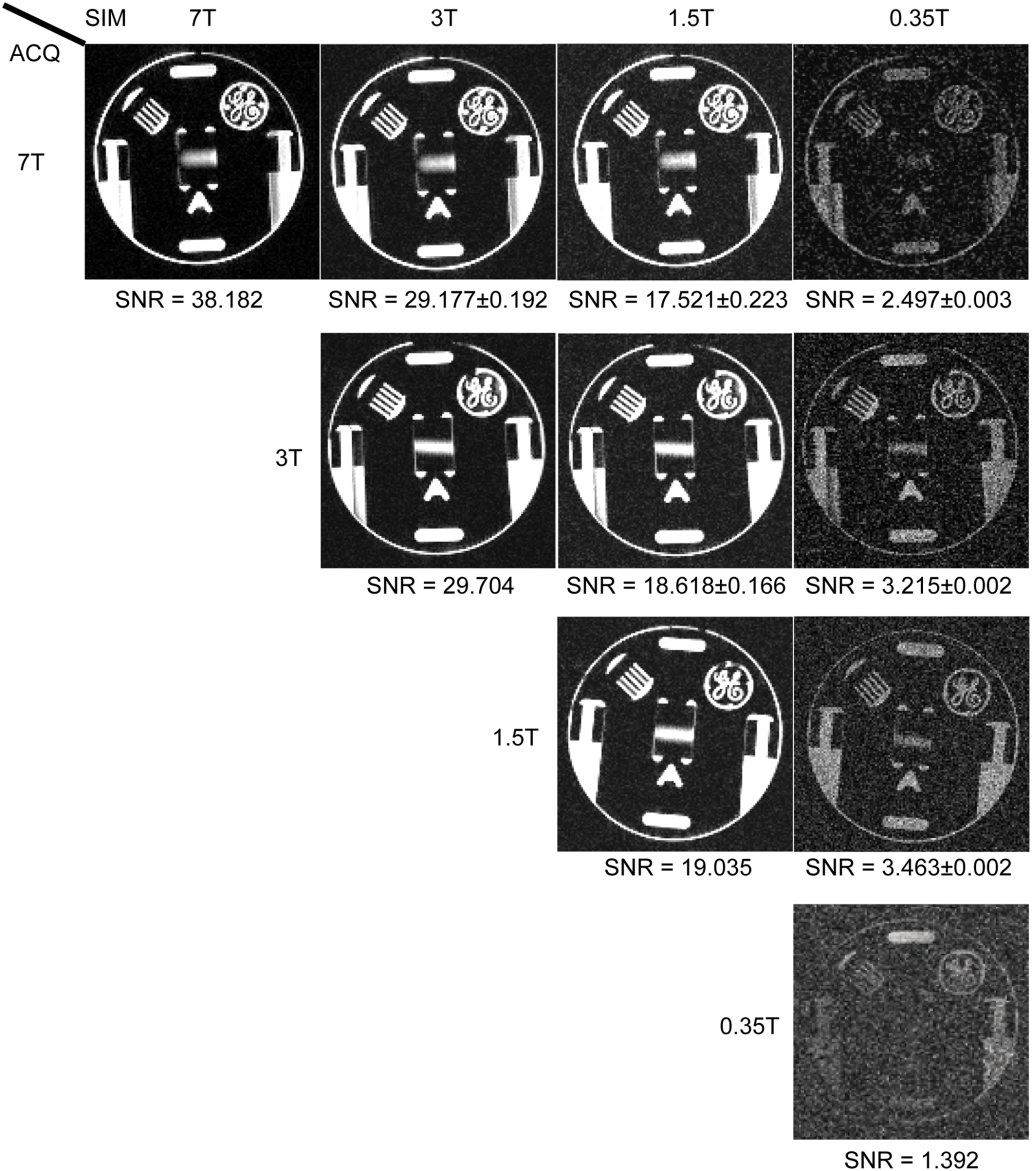
Phantom Validations of Simulated SNR Change. The acquired 0.35T/1.5T/3T/7T images and simulated images from data acquired at 3T and 7T respectively are listed for comparison. Measured SNR values are shown below each image. For simulated images, the mean and standard deviation of SNR of twenty different simulations were used. Contrast was adjusted for better noise visualization.

Fig 2 compares the image acquired and/or simulated at 0.35T, 1.5 T, 3 T, 7 T. The difference between simulated and measured mean SNR was less than 8% for all images at 1.5 T and 3 T respectively, which was considered to be good agreement. There were a 1.8–2.5 times of SNR differences for simulated and acquired images at 0.35T.

### Real-time Upper Airway Imaging

**Fig 3:**
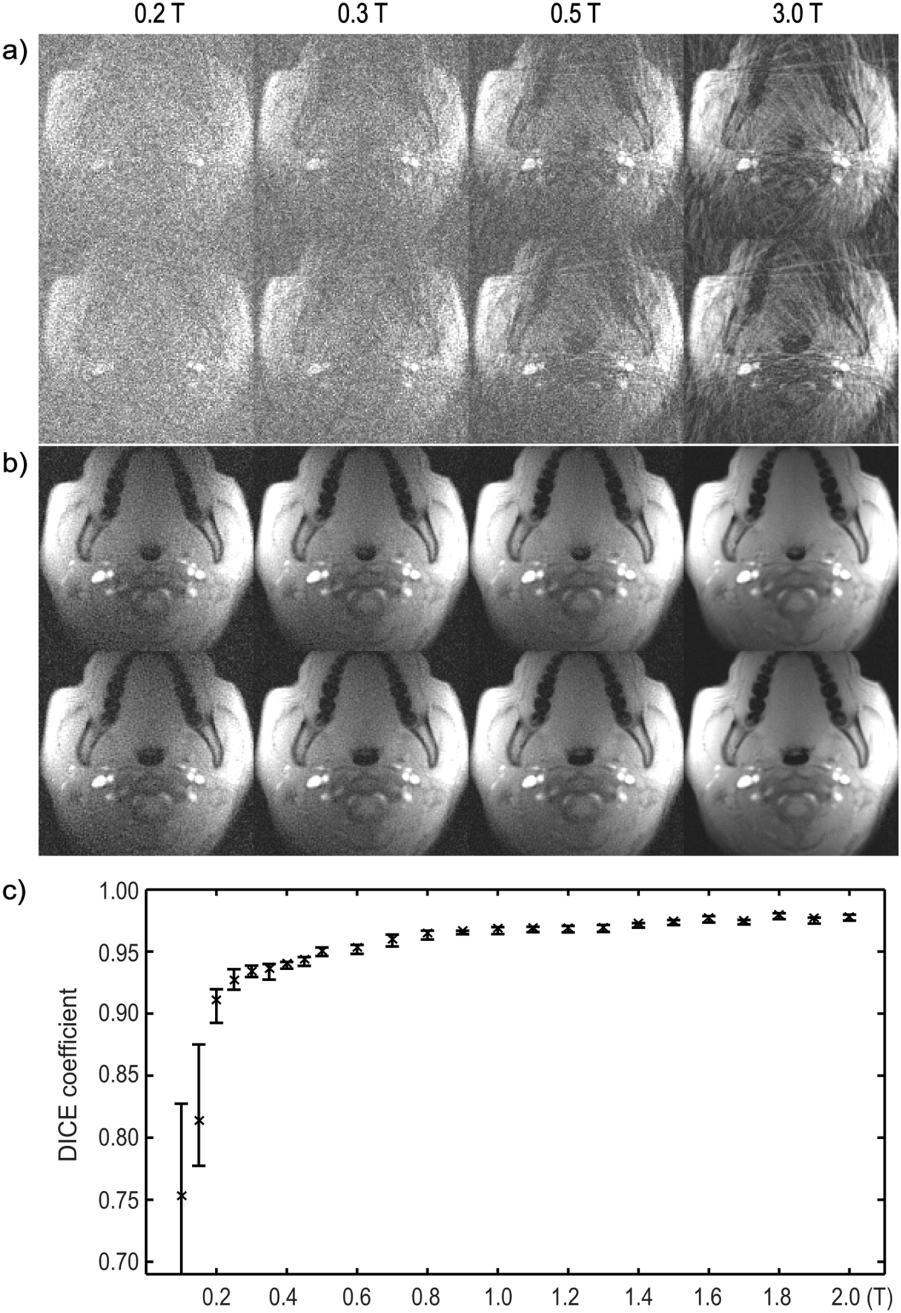
Application to Upper Airway Compliance Measurement. a) Gridding reconstruction for data acquired at 3 T & simulated at low field strengths. Two temporal frames are shown: one with the airway partially collapsed (top row) and one with it open (second row). Notice the strong noise that makes the airways gradually unidentifiable as field strength goes down. b) The same frames using CG-SENSE with temporal finite difference sparsity constraint. c) Airways segmented from images using reconstructions in b) are used to calculate the average DICE coefficients over 100 temporal frames (3 breaths) at different field strengths. 3T images are served as references. Fifty independent simulations were performed at each field strength. Error bars correspond to 95% confidence intervals.

Fig 3 shows two representative frames reconstructed at different field strengths, one with the airway partially collapsed (top rows in both a and b), and one with it open (bottom rows). Fig 3a and 3b correspond to gridding and CG-SENSE with temporal finite difference constraint, respectively. All reconstructed frames are also shown in the S1 Movie. The SNR becomes worse as field strength goes down, and the airway becomes completely unidentifiable below 0.3 T. With more advanced reconstructions in b, the noise and artifacts are reduced significantly. We then performed airway segmentation on these images based on a simple region-growing algorithm and show, in Fig 3c, the average DICE coefficients over 100 temporal frames (3 breaths) at different field strengths. Segmented airways from the 3T images were used as the references. Fifty independent simulations were performed at each simulated field strength. Error bars correspond to 95% confidence intervals. In our experience, DICE coefficient > 0.9 is acceptable for this application, suggesting that the minimum field requirement is 0.2 T. Note also that the DICE coefficients exhibit a sharp drop at 0.2 T and the variance increases significantly, implying segmentation failures.

### Fat-Water Separation

**Fig 4:**
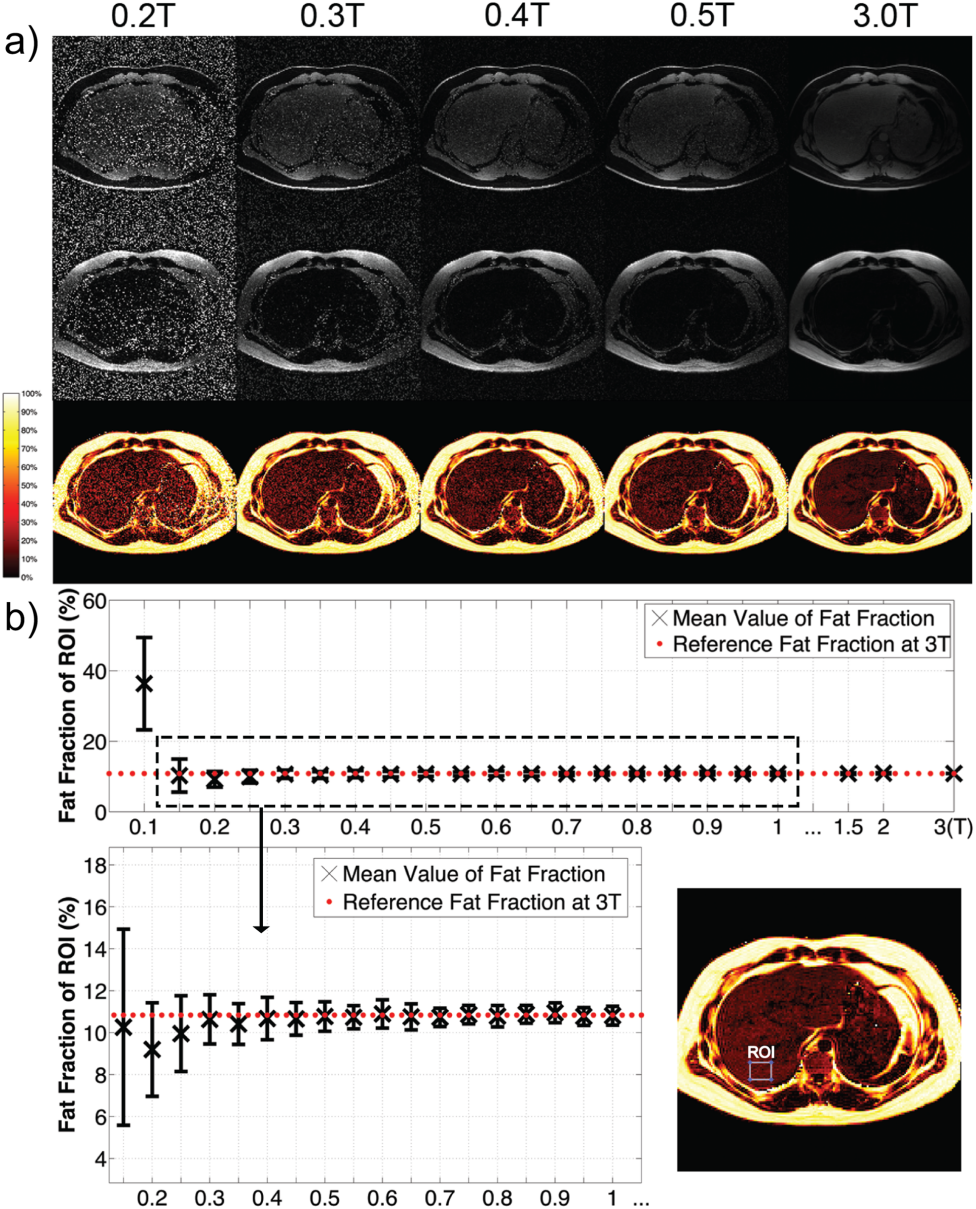
Application to Abdominal Fat-Water Separated Imaging. a) Fat-water separated images reconstructed from data acquired at 3 T and simulated at low fields. Top row: water only; middle: fat only; bottom: proton-density fat fractions. b) The mean and standard deviation of fat fraction in the ROI at different field strengths. Fifty independent simulations were performed at each field strength.

Fig 4a compares water-only, fat-only, and proton-density fat fraction images for a single axial slice at different field strengths. A region of interest (ROI) in the liver was manually selected and Fig 4b shows the mean and standard deviation of the fat fraction inside the ROI, calculated from fifty independent simulations at each field strength. The precision (standard deviation) becomes worse as B_0_ decreases. The accuracy (mean) deviates significantly at 0.1 T, a result of dominant noise making proton-density fat fraction biased towards 50%. Although the accuracy and precision needed for a clinical liver fat biomarker is not yet known [12], once determined, this type of analysis could facilitate determination of the required minimum field strength. For example, if the accuracy and precision needed are both 2%, then this analysis suggests a minimum field strength of 0.3 T would be sufficient.

## Discussion

It would be ideal to validate the assumptions and methods on a low-field scanner with very similar geometry, RF coils and sequence implementations as the high-field scanner. At the present time, most commercially-available low-field systems (<0.5T) are equipped with weaker, unshielded gradients, and a variety of other cost-cutting measures implemented. This makes it extremely difficult to make fair comparisons, and predict the performance of a low-field system with all other sub-systems intact. To eliminate the impact of these factors, we first performed the validation experiment using 1.5T, 3T, and 7T scanners from the same manufacturer with similar geometry and RF coils. To reduce the effects of B1 inhomogeneity and off-resonance, which are particularly severe at 7T, we used a small flip angle (10°) and short TE (3.1 ms) relative to T2 (100 ms) so that the validation can mostly reflect the accuracy of the assumptions and methods in this work. The phantom validation results exhibit a good match between the simulations and measurements on these scanners. It demonstrates that the assumptions we made are reasonable and the simulations based on them can give reliable predictions. We also performed a phantom experiment on a 0.35T scanner with different geometry and from a different vendor, with additional constraints on the RF receiver coil. The 1.8- to 2.5-fold SNR discrepancy between measurement and simulation at 0.35 T may be explained by unaccounted differences in the receive coils. The 0.35T ViewRay system is designed primarily for MRI-guided radiation therapy, therefore receiver coils are designed to minimally obstruct the desired radiation.

The proposed simulation framework has several limitations. First, because it is difficult to differentiate species with different relaxation properties in k-space, the framework only deals with PDw images, which significantly limits the number of real world applications. Second, some phenomena that were assumed to be independent of field strength, do actually change with field strength. For example, B_0_ and B_1_ uniformity are significantly improved at low-field, but the spatial distribution and the amount of the improvement is object specific. Phenomenon like physiological noise, must be experimentally studied and are extremely applications specific. In our experience, these factors would be nearly impossible to incorporate into a general-purpose simulator. Therefore, what we chose is to make simplifying assumptions that represent a worst-case scenario in most cases. For example, we assume the same B1 homogeneity at low-field even though the B1 transmit and receive field will have equal or better homogeneity at low-field. Third, image quality at low field can be optimized by adjusting pulse sequence parameters (e.g. using higher flip angles, longer readouts and TR’s with a larger readout duty-cycle). This was not accounted for, as we assumed pulse sequence parameters were unchanged. This again represents a worst-case scenario. Fourth, we performed a rather narrow validation experiment with just one resolution phantom. This was purely for practical reasons; it was the only common phantom available at all sites. Future experiments would benefit from a phantom that included samples with different PD and relaxation parameters (e.g. a T1/T2 grid phantom) and potentially in vivo data collection, using multiple sequences at each field strength.

Many new MR imaging and reconstruction methods are developed at centers that utilize state-of-the-art high-field instruments. In addition to advancing performance at high field strengths, it is informative to determine the potential to apply these same techniques on more affordable low-field systems. New methods, if translated and implemented on low-field scanners, could enable many applications that are now prohibitive at low field because of insufficient SNR. It is our experience that most MRI researchers today only have access to 1.5T/3T instruments, because they are the most widely used in the clinic. There are often significant logistical barriers to test ideas at low field. Getting an MR scanner, even a low field one, is a significant investment and the proposed simulation framework can provide a first-order approximation of performance and feasibility, at no cost.

Low-field MRI has the potential to be more cost-efficient, and has many other attractive properties including reduced acoustic noise and specific absorption rate (SAR), safer for subjects with implants containing ferromagnetic components, more uniform RF transmission, and less off-resonance for the same part-per-million B_0_ homogeneity. Lower RF frequencies also lead to decreased tissue conductivity and therefore higher RF transmission and reception efficiency, as quantitatively measured in [25,26]. If the effective SNR can be improved to reasonable levels with the help of better RF coil design, advanced imaging and reconstruction techniques, these nice features could further broaden the role of low-field MRI.

It is relatively straightforward to determine the minimum field strength requirement for new MRI methods under certain circumstances, as listed in Table 1. We have demonstrated the process, using modeling assumptions that are widely accepted in the MR community. However, several precautions need to be taken before applying the model here. First, the model assumes the same sequence parameters at all field strengths. It would be natural to pick different parameters at low fields. Second, the model assumes the same scanner geometry and coil geometry. This is also not perfect, since many design constraints change at low fields and they all could impact the magnet and coil layout. Third, the receiver coil noise goes down more slowly than the body noise as field strength goes down [27]. The validity of body thermal noise dominance is questionable for ultra-low field (< 0.1 T) and small volume imaging. Even in the range of 0.1 to 0.5 T, the requirement for suppressing receiver coil noise, although already achievable, is typically higher compared to at high fields. Finally, to achieve reasonable image quality at low fields, constrained reconstruction methods are likely to be involved in many applications. Although powerful, many of these methods have not been extensively validated yet. One needs to be extra careful with them, especially when the depiction of subtle features is important.

We would like to emphasize that due to the nature of MRI, poorer image quality is inevitable at low fields in almost all cases no matter what acquisition and reconstruction techniques are used. But as already been illustrated here and shown in several other low field studies [4,28–33], worse image quality does *not* necessarily lead to less diagnostic value. With that in mind, selecting appropriate evaluation criteria becomes very important when comparing the results at different field strengths. If, for example, the sensitivity and specificity of the useful features are comparable at both high and low fields, then differences in root-mean-square error (RMSE) are likely to be inconsequential.

## Appendix

### Signal relaxation corrections for common sequences

#### Spin echo (SE/FSE/TSE)

At steady state, the magnetization after excitation can be expressed as

$$M_{ss}\;=\;M_{0}\frac{1-E_{1}}{1-E_{1}cos\theta }sin\theta$$ (A1)

Where *M*_0_ is the longitudinal magnetization, *θ* is the flip angle and *E*_1_ = *e*^−*TR*/*T*1^. The acquired signal is

$$s=A\frac{\left(1-E_{1}\right)sin\theta }{1-E_{1}cos\theta }e^{-TE/T2}$$ (A2)

where A is a constant proportional to $B_{0}^{2}$. Because T_2_ is largely independent of field strengths that are commonly used in clinical MRI (0.1T–3T) [27,34], we neglect the differences of transverse relaxation due to T_2_ change at different field strengths (separate T2 values can always be measured when it goes to higher fields). According to Eqn. [4], the relaxation correction function becomes

$$f=\frac{{\hat{s}}_{l}}{a^{2}s_{h}}=\left[\frac{\left(1-E_{1,l}\right)sin\theta_{l}}{1-E_{1,l}cos\theta_{l}}/\frac{\left(1-E_{1,h}\right)sin\theta_{h}}{1-E_{1,h}cos\theta_{h}}\right]\;e^{-\left(TE_{l}-TE_{h}\right)/T2}$$ (A3)

*l* and *h* stand for low and high field respectively. For PDw imaging, where a) TE << T_2_ and b) *TR >>* T_1_ or the flip angle *θ* is low, *f* ≈ *sinθ*_*l*_ / *sinθ*_*h*_ regardless of the species type. As a result, the restriction of single species dominance can be relaxed and the equation above can be applied to multiple species types.

#### Gradient echo (GRE/FGRE/SPGR/FLASH)

The signal change is similar to spin echo, except following T_2_* decay:

$$f=\frac{{\hat{s}}_{l}}{a^{2}s_{h}}=\left[\frac{\left(1-E_{1,l}\right)sin\theta_{l}}{1-E_{1,l}cos\theta_{l}}/\frac{\left(1-E_{1,h}\right)sin\theta_{h}}{1-E_{1,h}cos\theta_{h}}\right]e^{-\left(TE_{l}/T2_{l}^{*}-TE_{h}/T2_{h}^{*}\right)}$$ (A4)

In practice, one may not know the explicit values of T_2_*, since it also depends on local B_0_ inhomogeneity and susceptibility. Given [35]

$$\frac{1}{T2^{*}}=\frac{1}{T2}+\text{c}\gamma \Delta B_{ppm}B_{0}$$ (A5)

where c is a constant and Δ*B*_*ppm*_ is the field inhomogeneity in parts-per-million. We can rewrite the exponential term in (A4) as:

$$e^{-\left(TE_{l}/T2_{l}^{*}-TE_{h}/T2_{h}^{*}\right)}=e^{-\left(TE_{l}-TE_{h}\right)/T2}e^{-c\gamma \Delta B_{ppm}\left(B_{0,l}TE_{l}-B_{0,h}TE_{h}\right)}$$ (A6)

In cases where T_2_* is difficult to estimate, T_2_ may be used instead of T_2_*. As long as *B*_0,*l*_*TE*_*l*_ ≤ *B*_0,*h*_*TE*_*h*_, this will lead to an underestimation of signal, which means the simulated SNR will be at best the same as the actual low-field acquisition.

In the airway example, T_2_* is unknown, so T_2_ is used instead. Given proton density weighting and *θ*_*l*_ = *θ*_*h*_, *f* ≈ 1. In the fat-water example, in order to generate the same fat-water phase shift, the product of B_0_TE needs to remain the same, (A6) is reduced to $e^{-\frac{TE_{l}-TE_{h}}{T2}}$. Since small flip angles *θ*_*l*_ = *θ*_*h*_ = 3° were used, $f\approx \;e^{-\frac{TE_{l}-TE_{h}}{T2}}$, with liver T_2_ set to 42 ms [27].

#### Balanced steady-state free precession (bSSFP, FIESTA, true FISP)

The steady state transverse magnetization, assuming TE, TR *<<* T_1_, T_2_, is [36]:

$$M_{ss}\;=\;M_{0}\frac{sin\theta }{1+cos\theta +\left(1-cos\theta \right)\left(T1/T2\right)}$$ (A7)

based on similar calculations in a), *f* is now a function of T_1_, T_2_ and flip angle:

$$f=\left(\frac{1+cos\theta +\left(1-cos\theta \right)\left(T1_{h}/T2\right)}{1+cos\theta +\left(1-cos\theta \right)\left(T1_{l}/T2\right)}\right)$$ (A8)

#### Inversion recovery (STIR, FLAIR)

Following similar analysis, with 90° excitation, we have:

$$M_{ss}\;=\;M_{0}\left(1-2e^{-TI/T1}+E_{1}\right)$$ (A9)

$$f=\left[\left(1-2e^{-TI_{l}/T_{1,l}}+E_{1,l}\right)/\left(1-2e^{-TI_{h}/T_{1,h}}+E_{1,h}\right)\right]e^{-\left(TE_{l}-TE_{h}\right)/T2}$$ (A10)

Since the inversion time *TI* is usually chosen to null a particular species, the impact of this species on the signal can be neglected. Here T_1_ is the value of the remaining dominant species.

## Supporting Information

S1 Movie Upper Airway Dynamics. The movie shows the whole 100 frames (3 breaths) reconstructed from simulated 0.2 T, 0.3 T, 0.5 T and acquired 3 T data (from left to right), as mentioned in Fig 3. The region around the airway is zoomed in for better illustration purpose. Top row: gridding reconstruction; bottom row: CG-SENSE with temporal finite difference sparsity constraint.
(MP4)
